# Deep learning pipeline for trapezium segmentation in thumb radiographs

**DOI:** 10.1186/s41747-026-00678-2

**Published:** 2026-02-23

**Authors:** Victor Maigné, Youssef Frikel, Félix Barbier, Mélanie Courtine, Younes Bennani, Thomas Grégory

**Affiliations:** 1 Orthopaedic Surgery Department, Hôpital Avicenne, AP-HP, Université Paris Sorbonne Nord, Bobigny, France; 2 Maison des Sciences Numériques, Université Paris Sorbonne Nord, Villetaneuse, France

**Keywords:** Arthroplasty, Artificial intelligence, Deep learning, Radiography, Thumb

## Abstract

**Objective:**

Accurate identification of the trapezium is crucial for trapeziometacarpal (TMC) arthroplasty but remains challenging on standard radiographs due to overlapping anatomy. Artificial intelligence has shown promise in musculoskeletal imaging, yet its application to small joints is limited.

**Materials and methods:**

We retrospectively analyzed 624 thumb radiographs, of which 519 met the inclusion criteria. Radiographs of insufficient quality—blurred images or non-centered TMC joints—were excluded by consensus of two hand surgeons. Manual trapezium annotations performed by an expert surgeon were reviewed by two additional surgeons. Inter-observer agreement was assessed on 10% of cases using Cohen κ. We developed a two-stage deep learning pipeline combining You Only Look Once (YOLO)v8 for trapezium detection with U-Net for segmentation. Its performance was compared with the standalone U-Net, segment anything model (SAM), and Mobile-SAM. Detection accuracy was measured using mean average precision (mAP), while segmentation was evaluated with Dice similarity coefficient (DSC) and intersection over union (IoU).

**Results:**

YOLOv8 achieved a detection mAP of 99.5%. The combined YOLOv8 + U-Net model yielded a DSC of 94.2% and an IoU of 89.1%, outperforming U-Net (DSC 89.5%, IoU 81.2%), SAM (Dice 88.8%, IoU 80.3%), and Mobile-SAM (Dice 88.9%, IoU 80.5%). Inter-observer agreement was excellent (κ = 0.89, DSC = 93.8%).

**Conclusion:**

The proposed two-stage pipeline provides accurate, reproducible trapezium segmentation on radiographs, outperforming widely used models. This approach may enhance preoperative planning and intraoperative guidance in TMC arthroplasty.

**Relevance statement:**

This two-stage AI pipeline enables precise trapezium segmentation on thumb radiographs, supporting improved surgical planning and intraoperative guidance in TMC arthroplasty, with potential to enhance implant placement accuracy and patient outcomes.

**Key Points:**

- A two-stage AI pipeline (YOLOv8 + U-Net) accurately detects and segments the trapezium on thumb radiographs.
- The method outperforms popular segmentation models and achieves expert-level reproducibility.
- This tool may enhance surgical planning and intraoperative guidance for TMC arthroplasty.

**Graphical Abstract:**

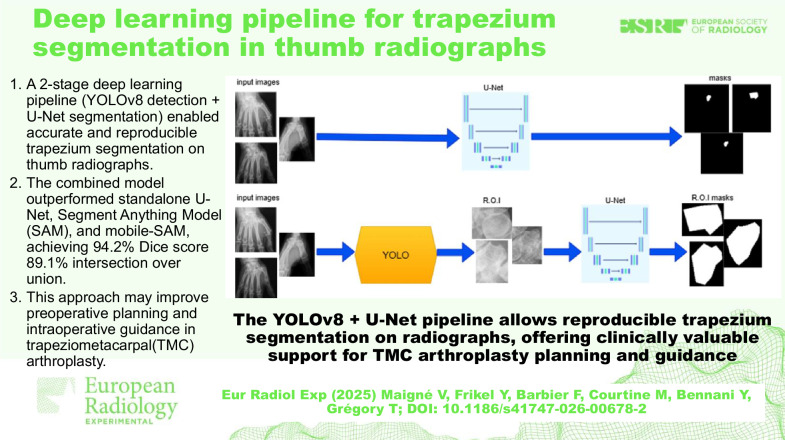

## Background

Osteoarthritis of the trapeziometacarpal (TMC) joint, or rhizarthrosis, is a common degenerative disorder, particularly in postmenopausal women [1, 2]. When conservative management fails, TMC arthroplasty offers effective pain relief and functional recovery but remains technically demanding [3, 4]. Precise preparation of the trapezium and implant positioning are crucial for long-term stability.

Radiography is the mainstay for diagnosis and preoperative planning, but trapezium visualization is often hampered by overlapping structures and variations in morphology [5–7]. Artificial intelligence (AI) has recently shown strong potential in musculoskeletal radiology [8, 9], particularly for fracture detection and large joint arthroplasty planning [10–13]. Automated bone segmentation has already facilitated implant templating in total knee and hip arthroplasty. However, to date, few applications have focused on small joints such as the thumb, where radiographic challenges are greater, and implants are smaller [12, 13].

A recent study [14] introduced a two-stage deep learning model for femoral segmentation, demonstrating the superior accuracy of cascaded detection–segmentation approaches. Inspired by this, we developed a similar two-stage pipeline for the trapezium. We hypothesized that combining You Only Look Once (YOLO)v8 detection with U-Net segmentation would outperform traditional segmentation networks and provide clinically relevant support for both preoperative and intraoperative use.

## Materials and methods

### Study population and dataset

**Fig. 1:**
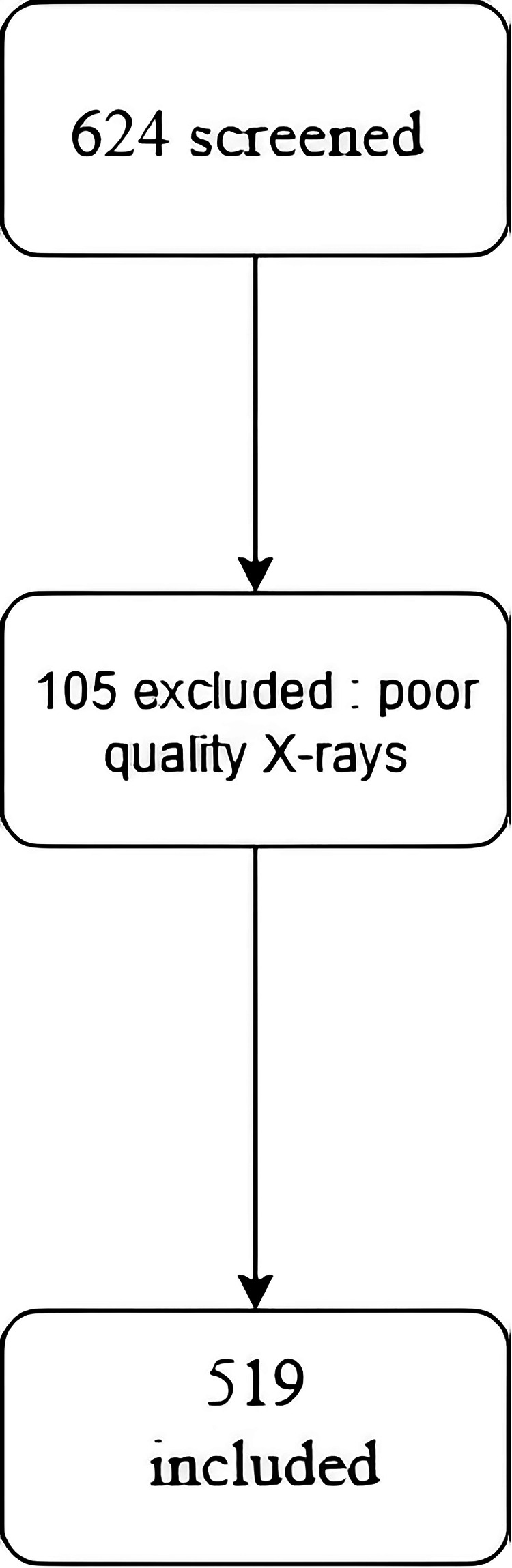
Study flow-chart

This retrospective single-center study included 624 consecutive thumb radiographs acquired between January 2015 and December 2022 at Hôpital Avicenne (Assistance Publique-Hôpitaux de Paris, Bobigny, France) (Fig. 1). After applying exclusion criteria—such as incomplete visualization of the trapezium, motion artifacts, or advanced trapezial collapse causing non-identifiable anatomy (see Fig. 1)—519 radiographs from 412 patients were retained for analysis. The study was approved by the local institutional review board (Approval #CLEA-2025-434), and the requirement for written informed consent was waived owing to the retrospective, anonymized design. Radiographs were acquired as part of standard clinical care, primarily in anteroposterior and lateral projections.

### Reference standard

Manual annotations of the trapezium were initially performed by an expert hand surgeon using the open-source software *Labelme* (MIT Computer Science and Artificial Intelligence Laboratory). These annotations were subsequently reviewed and validated by two additional independent hand surgeons. Discrepancies were resolved by consensus discussion. To assess inter-observer reproducibility, a randomly selected subset of 10% of the dataset was independently annotated by another surgeon, and agreement was quantified using Cohen κ and Dice similarity coefficient (DSC).

### Deep learning pipeline

A two-stage deep learning pipeline was implemented. In the first stage, a YOLOv8 model (Ultralytics; https://www.ultralytics.com) automatically detected the trapezium region on each radiograph [10]. In the second stage, the detected region was input into a U-Net network to perform precise segmentation [11] (Fig. 3). This sequential approach was designed to improve localization accuracy and boundary delineation by focusing the segmentation network on a region of interest rather than the entire radiograph. Data augmentation techniques—including random rotations (± 15°) and horizontal or vertical flips—were applied to enhance model robustness and mitigate overfitting.

The proposed two-stage model was compared with several established segmentation architectures, including standalone U-Net, SAM, and Mobile-SAM, all trained and validated on the same dataset [12, 13]. For comparison, we also tested a newer YOLO architecture (YOLOv11) for trapezium detection to assess potential performance differences with YOLOv8. The dataset was randomly split into 80% for training and 20% for testing, ensuring no patient overlap between subsets.

### Performance metrics

Detection performance was evaluated using mean average precision (mAP) [10] at an intersection-over-union (IoU) threshold of 0.5. Segmentation performance was assessed using the DSC and mean IoU between predicted and ground-truth masks [11]. Quantitative results were reported as descriptive statistics, without hypothesis testing, since the objective was to benchmark absolute model performance rather than to assess statistical differences.

### CLAIM framework compliance

This study adhered to both the checklist for artificial intelligence in medical imaging (CLAIM) [15] and the Standards for Reporting Diagnostic Accuracy Studies (STARD) [16] to ensure methodological rigor and transparency. Inclusion and exclusion criteria were explicitly defined, manual annotations were independently reviewed by multiple experts, and dataset partitions for training and testing were clearly reported. Reproducibility was assessed through inter-observer agreement metrics (Cohen κ and DSC). All preprocessing steps, model architectures, and evaluation methods were described in detail to facilitate replication and adherence to current best practices in AI-driven medical imaging research.

### Data analysis

Differences are reported descriptively; no statistical testing was performed because the objective of the study was to benchmark the absolute performance of each model rather than to test a specific statistical hypothesis. As recommended in methodological guidelines for AI model comparison, formal statistical testing is not required when the study does not aim to demonstrate superiority or non-inferiority, but rather to provide a descriptive performance evaluation.

## Results

### Study population

A total of 519 radiographs from 412 patients were included (Table 1). The mean patient age was 66.4 years (range 48–84), and 78% were women. Radiographs consisted of standard anteroposterior and lateral projections, with 56% right-hand and 44% left-hand images.

**Table 1:** Patient demographics (*n* = 412)

Characteristic	Value
Mean age, years (range)	66.4 (48–84)
Sex, female	78%
Number of radiographs	519
Standard projections	Antero-posteror 100%, lateral 85%
Side	Right 56%, left: 44%

### Detection and segmentation performance

YOLOv8 achieved a detection mAP of 99.5%. The combined YOLOv8 + U-Net pipeline achieved a DSC of 94.2% and IoU of 89.1%, outperforming standalone U-Net (DSC 89.5%, IoU 81.2%), SAM (DSC 88.8%, IoU 80.3%), and Mobile-SAM (DSC 88.9%, IoU 80.5%) (Table 2). YOLOv11 reached a detection mAP of 99.1%, with segmentation DSC of 89.0% and IoU of 81.1%, slightly lower than YOLOv8 + U-Net but superior to standalone U-Net and SAM-based models.

**Table 2:** Comparison of model performance for trapezium segmentation

Model	mAP (%)	IoU (%)	DSC (%)	Cohen κ
YOLOv8	99.5	75.3	84.9	‒
YOLOv11	99.1	81.1	89.0	‒
SAM	n/a	80.3	88.8	‒
Mobile-SAM	n/a	80.5	88.9	‒
U-Net	n/a	81.2	89.5	‒
YOLOv8 + U-Net	n/a	89.1	94.2	‒
Inter-observer agreement	‒	‒	93.8	0.89

*IoU* Intersection over union, *mAP* Mean average precision, *DSC* Dice similarity coefficient

### Inter-observer variability

Agreement between expert annotators was as follows: Cohen κ = 0.89; DSC = 93.8%.

### Qualitative assessment and critical observations

**Fig. 2:**
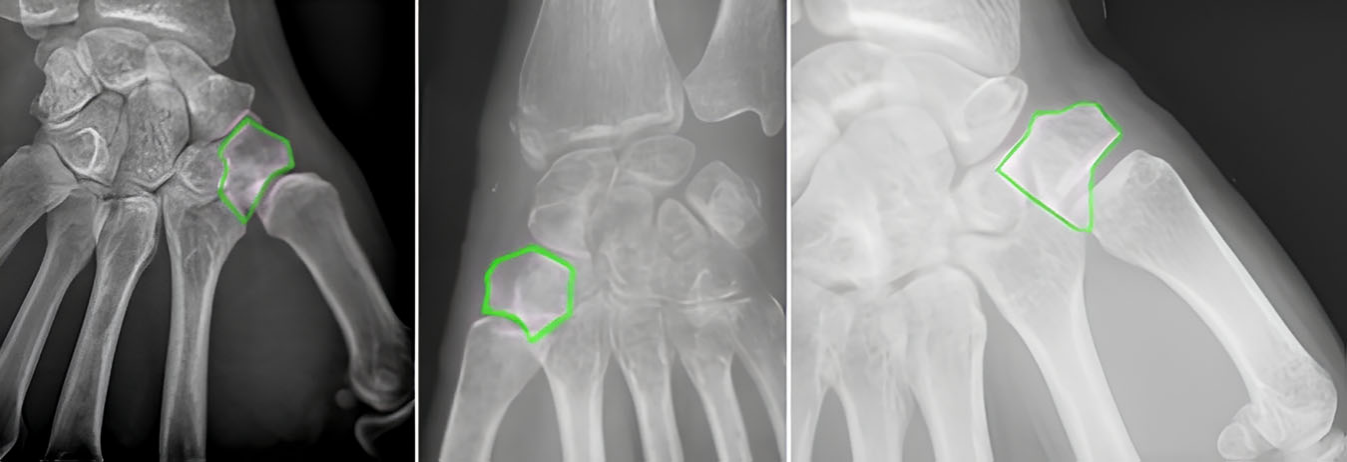
Radiograph from dataset (Hôpital Avicenne, Assistance Publique-Hôpitaux de Paris, Bobigny, France, anonymized image) showing the trapezium (green box). The bone is partially obscured by surrounding structures, highlighting the need for automated localization. Inter-observer agreement was excellent, with Cohen κ = 0.89 and DSC = 93.8%

**Fig. 3:**
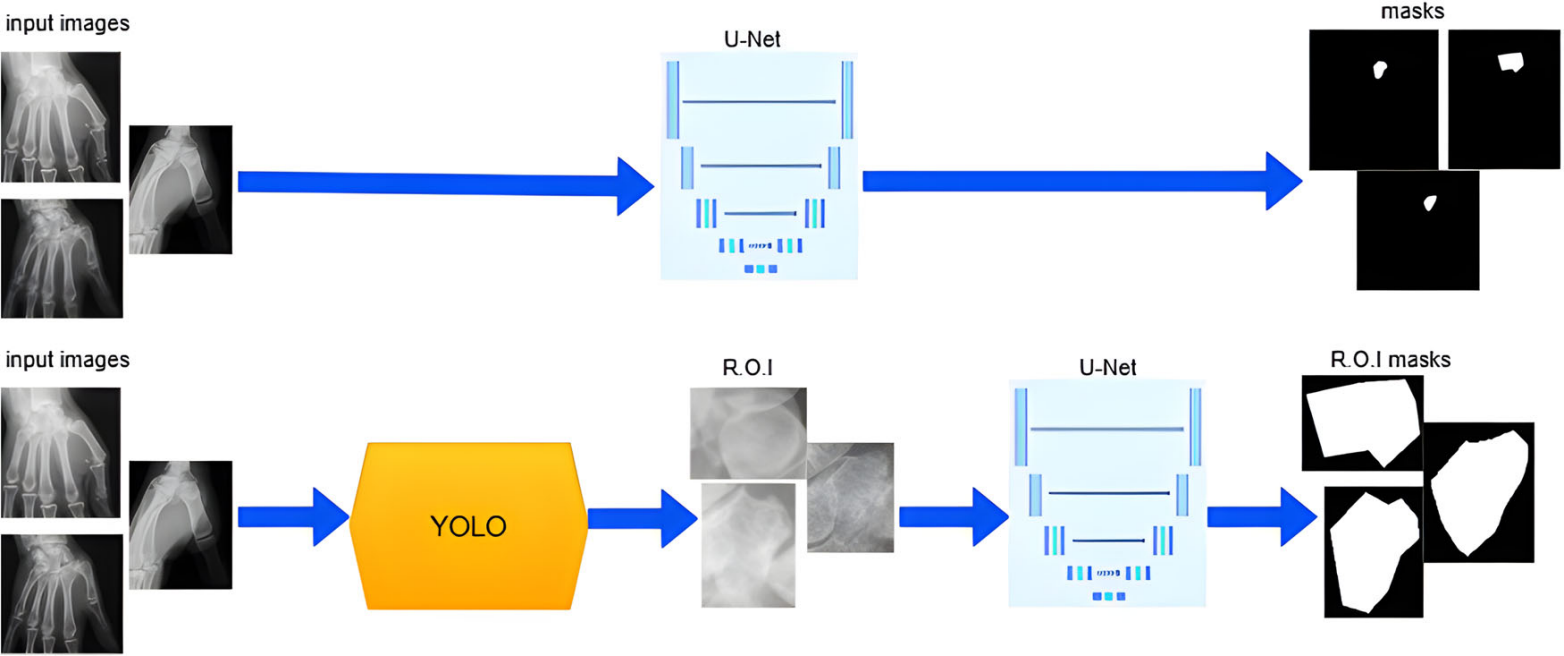
Illustration of the traditional full-image segmentation approach compared to the proposed two-step pipeline: detection-based region-of-interest feeding a segmentation network

**Fig. 4:**
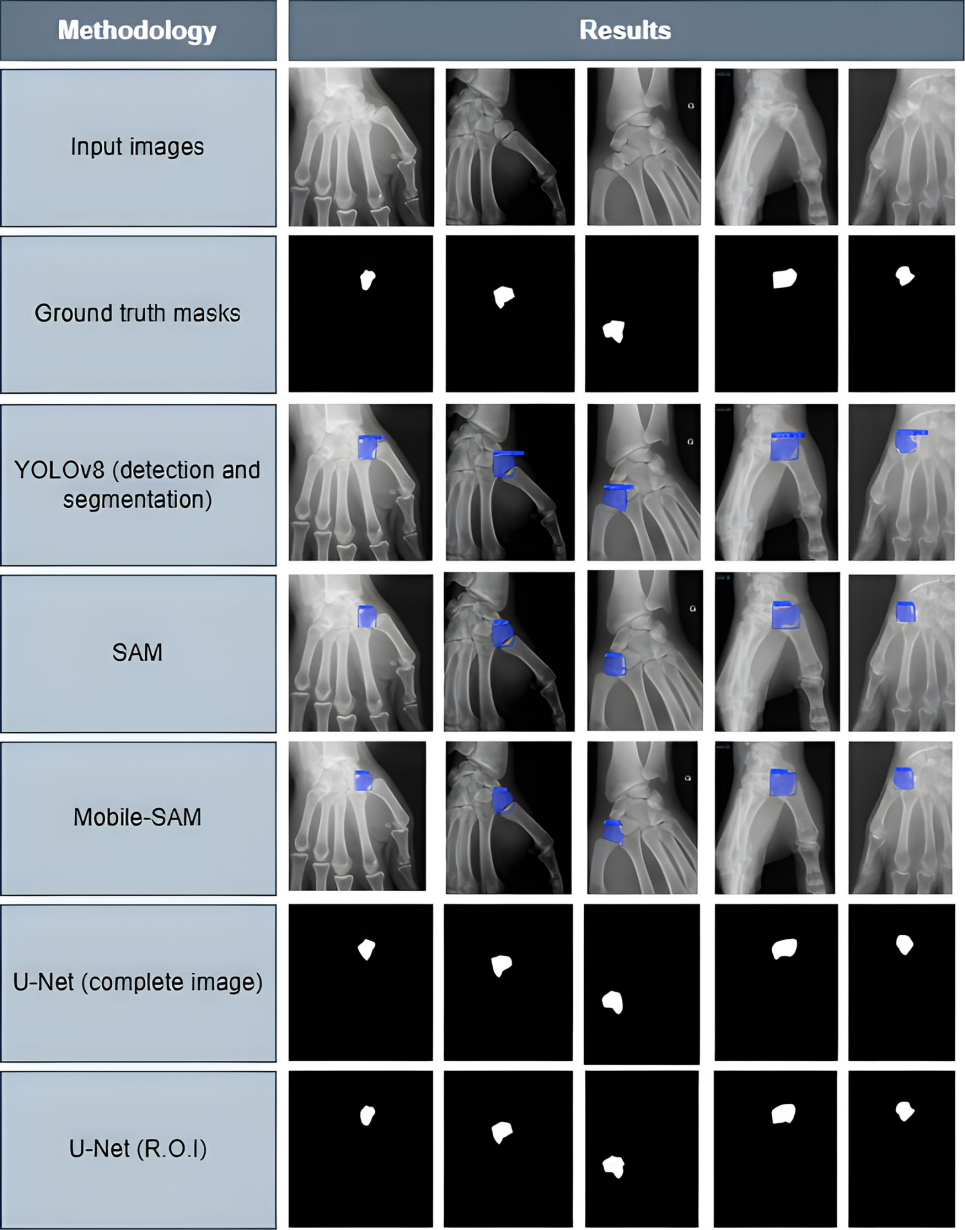
Segmentation results using different models. Ground truth masks are shown in red; predicted masks in green. YOLOv8 + U-Net demonstrates improved overlap and boundary accuracy

Representative examples of segmentation (Figs. 2–4) highlight the superior boundary delineation of the two-stage pipeline. Despite the high overall performance, challenges remain in lateral projections where adjacent metacarpal or carpal bones partially overlap the trapezium. Minor under- or over-segmentation was observed in complex anatomical regions, reflecting limitations of standard radiograph resolution. Compared with standalone models, the two-stage pipeline produced smoother, more contiguous masks and fewer spurious predictions, supporting both preoperative planning and potential intraoperative guidance. These observations emphasize the clinical relevance of the YOLOv8 + U-Net approach while identifying avenues for future refinement.

## Discussion

This study demonstrates that a two-stage deep learning pipeline combining YOLOv8 detection with U-Net segmentation can reliably identify and segment the trapezium on standard thumb radiographs. The two-stage approach consistently outperformed standalone segmentation models, highlighting the benefit of a cascaded detection–segmentation strategy [10–13]. Inter-observer agreement was excellent (Cohen κ = 0.89), confirming the reliability of the reference annotations [3–7].

Automated trapezium segmentation has several potential clinical applications. Preoperatively, it allows surgeons to anticipate implant size, orientation, and positioning. Analogous to computer-assisted navigation in total knee arthroplasty, which reduces operative time as experience grows [17], AI-based guidance could streamline surgical workflows for TMC arthroplasty. Intraoperatively, AI-assisted visualization on fluoroscopy could accelerate the learning curve, minimize technical errors, and support precise cup placement, particularly during critical steps such as broaching and implantation. Accurate segmentation may decrease the risk of component malposition, loosening, or dislocation, paralleling outcomes reported with navigation-assisted knee arthroplasty [18, 19]. Integration with PACS would allow seamless overlay of AI-generated masks on clinical images, facilitating intraoperative decision-making.

Our results (DSC 94.2%, IoU 89.1%) are consistent with, or superior to, recent studies on small-joint segmentation. Moradmand et al [20] developed a multistage deep learning framework for joint localization in rheumatoid arthritis hand radiographs, achieving high accuracy across hundreds of subjects. Lien et al [21] used attention mechanisms to enhance segmentation in small joint regions under limited data conditions. Ponnusamy et al [22] demonstrated accurate finger joint segmentation while reducing manual annotation burden. These studies underscore the importance of robust model performance on small anatomical structures, strategies for handling limited datasets, and external validation, all of which informed our study design.

This study has several limitations. It was retrospective and conducted at a single center, potentially limiting generalizability. Radiographs originated from a limited number of imaging systems, and expert annotations, while highly reliable, may introduce variability. The model has not yet been tested in real-time intraoperative settings, where fluoroscopic positioning and image quality may vary. Prospective multicenter validation across diverse patient populations and imaging systems is warranted.

Future work should evaluate the proposed pipeline in prospective multicenter studies to confirm its generalizability across diverse imaging systems and patient populations. Integration of the model for real-time intraoperative guidance with automated feedback could support surgeons during critical steps such as broaching and cup placement. Additionally, predictive modeling of implant orientation and sizing could further enhance preoperative planning. Finally, assessment of the impact of AI-assisted guidance on operative time, complication rates, and revision frequency will be essential to determine the clinical benefit of this approach.

In conclusion, the two-stage YOLOv8 + U-Net pipeline provides accurate and reproducible trapezium segmentation, with strong potential for translation into clinical practice. By reducing variability and enhancing surgical precision, it may improve implant positioning and patient outcomes, bringing small joint arthroplasty closer to the standards of computer-assisted navigation already established in larger joints.

## Data Availability

The data supporting the findings of this study are available from the corresponding author upon reasonable request.

## Abbreviations

AI: Artificial intelligence
CLAIM: Checklist for artificial intelligence in medical imaging
DSC: Dice similarity coefficient
IoU: Intersection over union
mAP: Mean average precision
TMC: Trapeziometacarpal
YOLO: You only look once
